# South Africa's Experience of the Closure of the Cellulose Sulphate Microbicide Trial

**DOI:** 10.1371/journal.pmed.0040235

**Published:** 2007-07-31

**Authors:** Gita Ramjee, Roshini Govinden, Neetha S Morar, Anthony Mbewu

## Abstract

The researchers who conducted the cellulose sulphate microbicide trial share the lessons they learned from the trial's early closure.

In sub-Saharan Africa, almost 60% of HIV infections are among women [1], and the number of new HIV infections in women worldwide continues to escalate. The high incidence of HIV in many African countries provides the optimum environment for research on technologies that could prevent women from becoming infected, including microbicides. In this article, we discuss the recent highly publicised closure of a trial of cellulose sulphate (CS), which we conducted. We discuss the impact of the closure on the participants, the community at the trial site and the public at large, the public health sector, national regulatory bodies, the media, and on other ongoing microbicide trials. The local lessons that we learnt from the closure may provide guiding principles for researchers and advocates in the HIV prevention field as a whole, who may face similar situations in the future.

## Previous Microbicide Trials

Vaginal microbicides are products which, when applied to the vagina, may prevent HIV transmission. Such a product would be particularly valuable for women who are unable to negotiate condom use with their partners, since its use would be initiated by the woman. The concept of a vaginal microbicide was tested several years ago using an over-the-counter spermicide, nonoxynol-9, a surfactant that acts by disrupting cell membranes. The trial, conducted in several African countries, showed an increase in risk of HIV among women who used the product more than three times a day [2].

The trial outcome was a huge setback for the microbicide field. Nevertheless, almost a decade later, there are several products in large-scale clinical trials. These products were developed as a result of a better understanding of HIV-1 pathogenesis, including identification of HIV target cells [3].

In 2006, there was another disappointment with the stoppage of two trials of C31G (known as SAVVY), an antimicrobial and spermicidal agent. The trials were stopped because the HIV incidence was lower than expected in the target population, and it was unlikely that the trials would be able to show efficacy against HIV [4]. There were no safety concerns with the product.

Of the current products in large-scale effectiveness trials, almost all belong to a class of compounds called fusion inhibitors. These act by preventing the virus from attaching to the target cells in the vagina. The current generation of products has poor specificity to HIV. Two have contraceptive properties, three (BufferGel, Carraguard, and PRO 2000) have displayed in vitro evidence of inhibition of other sexually transmitted infections, and all of them are coitally dependent—that is, they must be used just prior to sexual intercourse [5].

**Figure 1:**
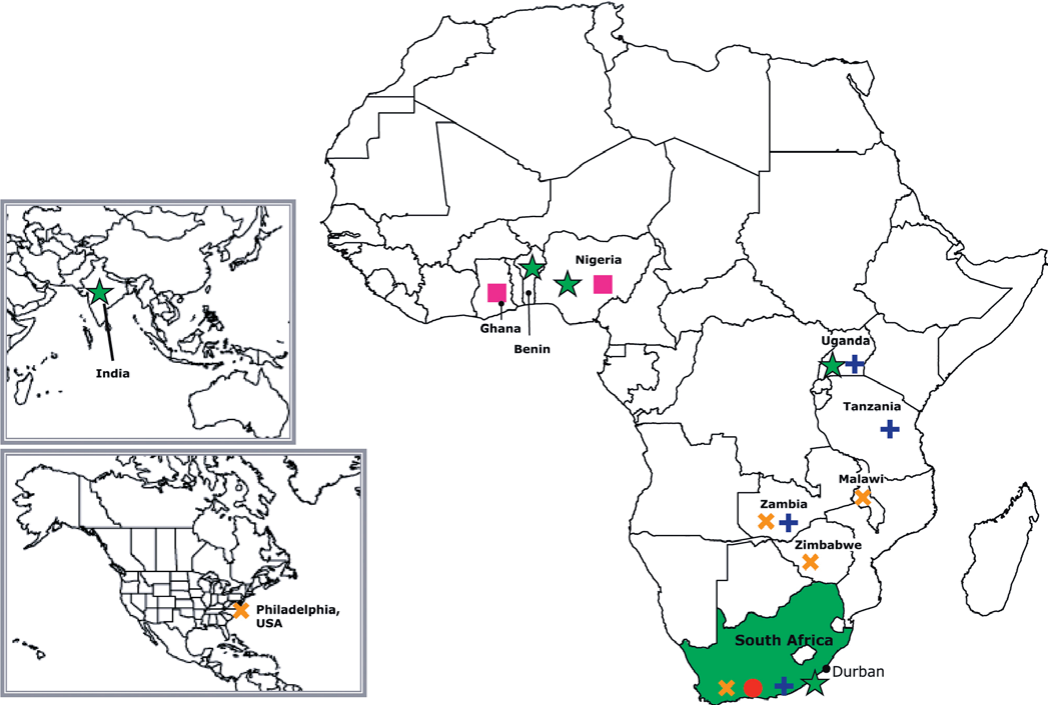
Global Phase IIb/III Microbicide Clinical Trials **Red circle:** Carraguard. Sponsored by Population Council. Phase III study of the efficacy and safety of the microbicide Carraguard in preventing HIV seroconversion in women. **Blue cross: 2% and 0.5% PRO2000.** Sponsored by Microbicide Development Programme. A phase III, multi-centre, randomised, double-blind, placebo-controlled trial to evaluate the effectiveness and safety of 0.5% PRO2000 and 2% PRO2000 gels for the prevention of vaginally acquired HIV infection. **Yellow cross: BufferGel and 0.5% PRO2000.** Sponsored by US National Institutes of Health. HPTN 035: Phase II/IIb safety and effectiveness study of the vaginal microbicides BufferGel and 0.5% PRO 2000 gel (P) for the prevention of HIV infection in women. **Green star: Cellulose sulphate—East and Southern Africa.** Sponsored by CONRAD. Randomised controlled trial of 6% cellulose sulphate gel and the effect on vaginal HIV transmission. **Green star: Cellulose sulphate—Nigeria.** Sponsored by Family Health International. Phase III trial of cellulose sulphate for HIV prevention. **Pink box: C31G (SAVVY).** Sponsored by Family Health International. Phase III trial of SAVVY in Ghana and Nigeria.

Until recently there were five products in large-scale phase IIb/III trials [6]: cellulose sulphate (Polydex Pharmaceuticals; http://www.polydex.com/) [7–11], PRO 2000 0.5% and 2% (Indevus Pharmaceuticals; http://www.indevus.com/) [12], Carraguard (Population Council; http://www.popcouncil.org/)[13,14], and BufferGel (ReProtect; http://www.reprotect.com/) [15]. Clinical trials of BufferGel and PRO 2000 are still ongoing in several parts of Africa (Figure 1). A clinical trial of Carraguard was completed in March 2007, and data analysis is in process. All ongoing clinical trials are reviewed regularly for safety by an external committee of experts—the data safety and monitoring committee (DSMC).

## Closure of the CS Trial

In early 2007, there was another huge disappointment. The randomised controlled trial testing 6% cellulose sulphate against a placebo gel for effectiveness against vaginal transmission of HIV, sponsored by the reproductive health research organisation CONRAD (http://www.conrad.org/), was stopped following recommendations by the DSMC after preliminary data review of 1,333 enrolled women from five sites (South Africa, Uganda, Benin, and two sites in India) suggested that there were more HIV seroconversions in the cellulose sulphate arm compared to the placebo arm of the trial. This unexpected outcome was a huge blow to the microbicide field as CS, a non-cyclic antimicrobial agent, had been tested in several safety trials previously and there were no concerns about safety based on these trials [16].

The study DMSC was requested to provide guidance to the investigators if data indicated a difference of *p* < 0.10 for futility or harm. At the first review of 1,333 women in late January 2007, there were 35 seroconversions from the three African sites, with a higher number of HIV seroconversions in the CS arm compared to the placebo arm. The interim data analysis suggested that the boundary for safety had been crossed, and so the DMSC recommended stopping the trial to ensure the safety of the participants [17]. Data analysis is still ongoing to ascertain the reasons why the product was found to be potentially harmful. Another trial of the same product at two sites in Nigeria did not show the same effect but was also stopped as a precautionary measure for the participants' safety [18].

Investigators at all sites were informed by CONRAD on 29 January 2007 of the trial closure and a press release was planned for 31 January 2007 [12]. The key message of the press release was that the trial was stopped because it was found that CS could lead to an increased risk of HIV.

## Actions, Challenges, and Responses to the Trial Closure

The HIV Prevention Research Unit (HPRU) of the Medical Research Council (MRC) in South Africa participates in all ongoing microbicide trials. Prior to the release of the press statement by CONRAD, we immediately developed a communication strategy to ensure that the information to stakeholders came from the local researchers (Table 1 and 2). Two days before the press release, we sent letters to the national and provincial departments of health, to the Medicines Control Council, which is South Africa's drug regulatory authority, and to the ethics committee that had approved the trial, informing them of the trial closure with a request for an urgent meeting.

**Table 1:**
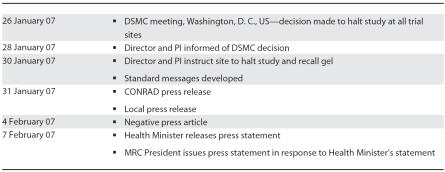
Key Dates

**Table 2:**
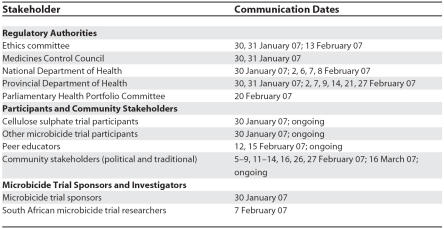
Communication with Regulatory Authorities, Participants, and Community Stakeholders

We also sent letters to all community partners advising them that there were new developments in microbicide research, and requested community meetings at local levels. These letters did not provide details of the outcome of the trial as we felt it was better to give these details in a larger community meeting. We made contact with non-governmental organisations, advocacy groups (such as the Gender AIDS Forum; see http://www.gaf.org.za/), women's groups, and, most importantly, the research participants of the CS trial itself (Table 1 and 2). Community outreach staff encouraged all participants to use the toll-free telephone numbers set up at all HPRU research sites for any questions and concerns. The vaginal gel (CS or placebo) was collected within one week from 80% of the women in the trial. Currently, 95% of the women have been successfully notified.

We contacted a journalist who writes regularly about HIV/AIDS issues, and asked her to write an article in a local newspaper providing information on the trial and reasons for its closure to avoid potential sensationalist reporting.

## Negative Press

Despite these proactive steps to inform the wider community, some reporters wrote inaccurate and sensational stories. For example, on 4 February 2007, a national weekly newspaper ran stories with the headlines “Medical research trial guinea pigs contract HIV” [19] and “Study to prevent AIDS left me infected”. These reports included sensational statements such as “Hundreds of women in South Africa, Benin, Nigeria, Uganda and India, who are being used as human guinea pigs in the US-funded research on HIV prevention, are feared to have contracted the virus during the course of the trials” [19]. In fact there were 35 sero-incident cases among 1,333 participants across all the African trial sites. This alarmist statement instilled fear amongst all trial participants. Statements in the article saying, for example, that women felt “used and misled” [19] falsely implied that the conduct of the study was unethical.

Subsequently, these sensational articles led to many spin-off articles in other local and national papers, including local language newspapers (Table 3). News stations were confused by the messages in these articles, and we were interviewed by several radio and television stations in order to clarify the concerns.

**Table 3:**
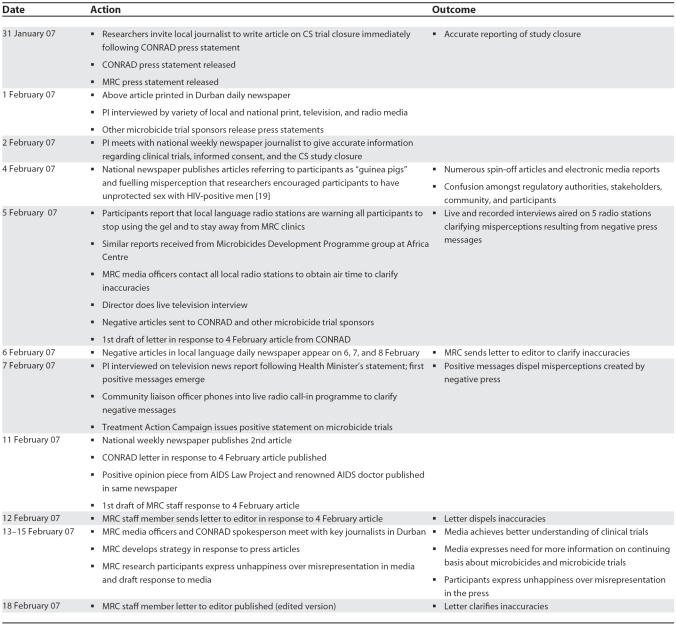
Communication with Media

The national Department of Health (DOH) requested a meeting with us to discuss the trial closure. Following this meeting, the minister of health, in consultation with her advisory committee, issued a press statement with the key message that all microbicide trials in South Africa would be investigated for ethical conduct [20]. This was the correct stance to take given the volatile situation, and we welcomed investigation of the trial conduct [21]. However, we believe it would have been an ideal opportunity to inform the larger population of the stringent and world-class ethical and regulatory standards that govern South African clinical trials. We believe that the DOH could have given a more balanced view of the situation. Such a view would have acknowledged that:

(1) the DOH is well informed of all clinical trials undertaken in the country through its clinical trial registry; (2) South Africa has national good clinical practice guidelines that must be followed for clinical trials; (3) all microbicide trials were conducted after thorough review of the protocols by ethics committees and drug regulatory authorities, with the latter governed by the DOH; and (4) these trials are regularly monitored by external reviewers.

Similar meetings on trial conduct were held with the local KwaZulu-Natal DOH and the Parliamentary Health Portfolio Committee, a parliamentary subcommittee of members of parliament tasked to address health issues.

## Impact upon Ongoing Microbicide Trials

Given that HPRU is involved in many clinical trials, the challenge was not only to address the closure of the CS trial, but to address the concerns of communities at trial sites of other ongoing microbicide trials in KwaZulu-Natal.

We had several meetings with political ward councillors, research communities, and other concerned stakeholders (Table 2). Communities misinterpreted the minister of health's press release, wrongly believing that the minister had called for all gel (microbicide) trials to be stopped (she had not—she had launched an investigation of the conduct of all microbicide trials). There were many irate people demanding answers to the following questions: (1) “Is it not unethical for researchers to ask innocent women to sleep with HIV-positive men so that we can test to see if the gel works?”; 2) “Is it true that gel increased the risk of HIV infection among innocent women?”; 3) “Why did researchers expose poor black women to the infected gel?”; and 4) “How did researchers explain the study to illiterate women?”

Clinical trial investigators in South Africa are required by the Medicines Control Council to reimburse trial participants with a minimum of R150 for trial participation to cover time, travel, and refreshments. But the general public's perception was that women were “bought to sleep with HIV-positive men”. Many people believed that the gel contained HIV or that simply inserting the gel increased the risk of HIV infection irrespective of the sexual act. Responses from trial participants such as “you infected us with HIV” gave credence to these misconceptions. Although the community entry, approval, and educational process of clinical trials was thorough at the start of all trials, there was confusion as many communities doubted the information given to them at the outset prior to trial implementation. Furthermore, some people expressed concern about the racial demographics of the trial participants, believing that we had “targeted” rural, poor, uneducated, and vulnerable women. The CS trial was in fact conducted at an urban site in Durban.

## Impact on Study Participants

Participants from all other microbicide trials were affected by closure of the CS trial. Male partners who knew about women's participation in other trials raised concerns that using “gel” increased HIV risk and did not want their female partners to participate in the trial. However, most women eventually decided to continue once they and their partners were counselled.

Peer educators in the community, who are also trial participants, were angry that the media described them as “poor”, “vulnerable”, “uneducated”, and “guinea pigs”. Women requested the researchers to link them to the media and the journalists who published inaccurate information so that they could voice their concerns. Most of the CS trial participants did not feel that trial participation increased their risk for HIV infection. They valued the benefit of being in the study. Less than ten of the CS trial participants believed the information in the press articles and were understandably upset. All except two participants agreed to speak and listen to staff, who allayed participants' fears. Two irate participants came to the clinic to return their gel and accused the researchers of trying to infect them with HIV. The partner of one participant burnt her gel supplies. Three participants and their partners attended the clinic for counselling.

**Recommendations for Communicating about HIV Prevention Trials**

- Emphasise community education.
- Explain and emphasise to the community that HIV seroconversion is the only way to measure effectiveness of new prevention technologies including microbicides (i.e., there are no surrogate markers of infection that can be used in trials).
- Educate the media and community about clinical trials, including regulatory procedures and good clinical practice guidelines followed by clinical trialists.
- Develop early drafts of press releases of all possible DSMC outcomes—positive, negative, and no effect—in partnership with local researchers and community representatives.
- Inform local ethics committees, drug regulatory authorities, and health authorities of trial outcome prior to press release.
- In drafting press releases, be sure to include the contribution of in-country investigators, community advisory boards, and other relevant bodies.
- Issue the press release in developing countries where the research is conducted. At the press conference, it is valuable to include the local principal investigator and representatives of the trial sponsor, ethics committee, and the local health authority.

## Lessons Learnt

The outcome of the nonoxynol-9 trial in 2000 was a huge setback for microbicide research in South Africa. Health authorities, ethics committees, and drug regulators were concerned about the safety of microbicides. Although the nonoxynol-9 trial received negative press, it was not as damaging at the community level as the closure of the CS trial, perhaps due to higher awareness now of microbicide clinical trials in South Africa as a whole. Furthermore, the 2006 closure of the SAVVY trial did not have an impact on current trials, possibly because the closure was not safety-related.

We learned several lessons from the closure of the CS trial that will provide us with a better understanding of communication strategies that may be required in many developing countries to deal with such situations in the future if they arise. Our recommendations for communicating about HIV prevention trials are shown in Box 1.

The first lesson we learned is that the phrasing of the CONRAD press release was open for misinterpretation by the lay public. Due to regulatory requirements of publicly traded companies such as Polydex Pharmaceuticals, which developed the CS microbicide, it was impossible for CONRAD to ensure that all sites were included in drafting the press release. We suggest that sponsors and in-country investigators be proactive and prepare communication strategies based on all possible outcomes of DSMC reviews whether they be positive, negative, or no effect. We also recommend that these potential messages be developed in consultation with local researchers, community advisory boards, or community representatives. Such advice from the community on shaping messages would help to reduce the risk of facts being distorted, and would help deliver the messages in a manner which is appropriate to the community's knowledge and understanding.

It is important that local health regulators such as the department of health and other governing bodies be kept informed on every aspect of the trial. Although regular updates were sent to the department, the frequency of updates was clearly not sufficient especially regarding negative outcomes of clinical trials. Quarterly meetings would ensure that the department is kept informed of all aspects of ongoing clinical trials. It is imperative that trial outcomes are reported to the department by local investigators prior to media release.

Similarly, the ethical review of clinical trials needs to be strengthened. Currently regulatory bodies approve clinical trials, but site reviews on the conduct of trials are limited, primarily due to lack of human capacity. It is important to boost the capacity of local ethics committees and other regulatory bodies to ensure that once the trials are approved, the sites are reviewed regularly so that there are no doubts created about trial conduct when there are unexpected trial outcomes.

For principal investigators (PIs), our experience provides a valuable lesson on the importance of ensuring involvement of communities in all aspects of research, including disseminating messages about clinical trial outcomes. Communities and participants should be kept updated on not only the trial in their community, but on outcomes of trials of other prevention technologies. Such open and transparent communication will improve the community's confidence in the researchers.

Fact sheets for communities need to be developed urgently once outcomes of prevention technologies are known. We have learnt that in addition to informing participant communities of the trial conducted in their community, it is important for us to provide them with an understanding of clinical trials in general. They need to understand that new drugs and interventions can only be introduced if the country's regulatory authority is convinced by the evidence of the quality, safety, and efficacy of the new product, and that such evidence can only come from clinical trials. They need to understand that clinical trials are particularly important if the product is designed for use by healthy individuals over a prolonged period of time, and that trials should preferably be conducted in communities that will use the product in case there are unforeseen pharmacogenetic interactions.

Perhaps the most important lesson learnt was that there is a need to educate the media on clinical trials as well as on the regulatory procedures and good clinical practice guidelines followed by clinical trialists. Despite our attempts to ensure that correct facts were published, the public was more attracted by sensational press articles. In most cases the media are hungry for news that will make headlines irrespective of whether the news is accurate or not. We suggest that sponsors and researchers set up media education sessions for each of the trials prior to implementation but also prior to results being released so that journalists have a good understanding of the outcomes of clinical trials and interpretation of data.

Since it is likely that most HIV prevention efficacy trials will be conducted in developing countries with high HIV incidence, we believe that the first press conference on a trial's results should be held in participating countries with the presence of the national principal investigator, a representative of the study's sponsor, and representatives from the local department of health and ethics committee. The in-country media would have the opportunity to direct any questions, concerns, or points of clarification to the principal investigator and sponsor rather than interpreting the results themselves. In our consultations with key journalists in Durban after the closure of the CS trial, they expressed the need to receive regular updates on microbicides and prevention research, rather than only receiving “bad” news about these trials.

One of the major challenges in HIV prevention research is that there are no surrogate markers for efficacy. The only way to assess effectiveness of products is to measure new HIV infections as an outcome. It thus becomes extremely difficult to make the lay public understand that in all prevention trials, participants are likely to become infected irrespective of the intervention, and it is not the researcher's aim to increase infection or risk of infection. Prevention packages are provided to avoid infection, including safe sex counselling, provision of male and female condoms, treatment of sexually transmitted infections, and intense scrutiny of safety markers such as ulceration and abrasions in vaginal microbicide trials in particular. Although such packages may reduce HIV incidence overall, it is our ethical imperative to provide as much preventive advice as possible to reduce the rate of new HIV infections. One of the lessons here is to make the broader community understand more clearly that the only way we can test effectiveness of an HIV prevention technology is to assess the number of new HIV infections.

## Conclusion

The closure of the CS trial has underscored the challenges we may face in the event of early trial closure due to a negative outcome. We now have insights on how to prepare for outcomes of future HIV prevention technologies and, at a minimum, prepare strategies to ensure that the messaging and process of message delivery is developed with local investigators, participant communities, local regulatory authorities, and in-country media.

We believe that the lessons learnt here will provide guidance to the HIV prevention field as a whole, as negative trial outcomes affect the future of HIV prevention research in the developing world.

## Abbreviations

CS: cellulose sulphate
DOH: Department of Health
DSMC: data safety and monitoring committee
HPRU: HIV Prevention Research Unit
MRC: Medical Research Council
PI: principal investigator
